# Supplementary material for: Understanding actions and challenges in protecting older people during covid-19 pandemic in indonesia: A qualitative study with female caregivers
Source: PLoS One. 2023 Mar 22;18(3):e0273761. doi: 10.1371/journal.pone.0273761 (PMC10032487; doi:10.1371/journal.pone.0273761)
Supplement: S1 File — (DOCX) [file pone.0273761.s001.docx]

Interview guide

1. Could you tell me your experiences as the main caregivers of your parents during COVID-19 pandemic?
2. What actions did you take to protect older people or parents during COVID-19 pandemic?
3. What challenges have you experienced when protecting older people during COVID-19 pandemic?
4. What are your main concerns when caring and protecting older people or your parents during pandemic?
5. What is your experience about older people’s adherence to the actions taken to protect them from COVID-19?
6. Could you tell me your experiences caring for and protecting your parents before COVID-19 pandemic and during pandemic? Is there any difference?
7. Is there anything else you would like to add?
