# Supplementary material for: Understanding actions and challenges in protecting older people during covid-19 pandemic in indonesia: A qualitative study with female caregivers
Source: PLoS One. 2023 Mar 22;18(3):e0273761. doi: 10.1371/journal.pone.0273761 (PMC10032487; doi:10.1371/journal.pone.0273761)
Supplement: Attachment — Submitted filename: Response to Reviewers.docx [file pone.0273761.s003.docx]

**Response to Reviewers**

Dear Editor,

Thank you very much for considering our manuscript **"** **UNDERSTANDING ACTIONS TAKEN BY FEMALE FAMILY CAREGIVERS AND CHALLENGES THEY FACED IN CARING FOR OLDER PEOPLE DURING COVID-19 PANDEMIC IN BELU DISTRICT, INDONESIA: A QUALITATIVE STUDY"** to be published in POLOS ONE Journal. Herewith we submit the revised version of our paper.

On behalf of all authors,

Sincerely,

Gregorius Abanit Asa

**RESPONSE TO THE JOURNAL**

**Comment**

Please ensure that you refer to Table 1 in your text as, if accepted, production will need this reference to link the reader to the Table.

**Response**

Table 1 has been referred in the text (in the result section prior to the table)

**Comment**

Please upload a copy of Figure 1 which you refer to in your text. Or if the figure is no longer to be included as part of the submission please remove all reference to it within the text.

**Response**

The copy of Figure 1 has been uploaded.

**Comment**

Please ensure that your manuscript meets PLOS ONE's style requirements, including those for file naming.

**Response**

The manuscript follows the PLOS ONE’s style requirements.

**Comment**

Please provide additional details regarding participant consent. In the ethics statement in the Methods and online submission information, please ensure that you have specified (1) whether consent was informed and (2) what type you obtained (for instance, written or verbal, and if verbal, how it was documented and witnessed).

**Response**

The study obtained the ethics approval from Health Research Ethics Committee, Duta Wacana Christian University, Yogyakarta, Indonesia (No. 1380/C.16/FK/2022). Each participant was informed about the aim of the study and there would be no consequences if they withdraw from the study without giving any reason. All participant signed and returned a written informed consent form via e-mail or WhatsApp a few days before the interviews. Each interview took 30-50 minutes and was recorded with the consent of the participants. Identification letter and number (e.g. R1, R2) was used for confidentiality purposes.

**Comment**

Please include a complete copy of PLOS’ questionnaire on inclusivity in global research in your revised manuscript. Our policy for research in this area aims to improve transparency in the reporting of research performed outside of researchers’ own country or community. The policy applies to researchers who have travelled to a different country to conduct research, research with Indigenous populations or their lands, and research on cultural artefacts. The questionnaire can also be requested at the journal’s discretion for any other submissions, even if these conditions are not met.  Please find more information on the policy and a link to download a blank copy of the questionnaire here: https://journals.plos.org/plosone/s/best-practices-in-research-reporting. Please upload a completed version of your questionnaire as Supporting Information when you resubmit your manuscript.

**Response**

Inclusivity in global research form has been filled (see supporting Information file)

**RESPONSE TO REVIEWERS**

**REVIEWER 1**

**Comment**

They need to add a brief explanation or justification about why they choose to conduct the study in Belu.

**Response**

Belu is reported as one of the districts in East Nusa Tenggara province with high number of COVID-19 cases, accounting 536 although it is a small district. Belu is selected because of small size, familiarity, and potential of undertaking the current study.

**Comment**

Data saturation: there seems to be a repetition of information about data saturation as it is mentioned in two places.

**Response**

This sentence “*The recruitment stopped when the authors felt the data saturation had been achieved”* is removed because it is a repetition.

**Comment**

Did face-to-face interviews follow COVID-19 prevention protocols? If yes, please make it clear in the methods section.

**Response**

Data were collected using in-depth interviews: face-to-face using masks and via telephone and zoom.

**Comment**

There is a supplementary file of COREQ checklist but you haven’t mentioned it in the text, please report it stating that you follow the guideline and why.

**Comment**

The study used consolidated for reporting qualitative studies (COREQ) to guide the report of the methods section of this study. The COREQ checklist contains 32 required items (Fig.1) for explicit and comprehensive reporting of qualitative studies especially interviews and focus groups.

**REVIEWER 2**

**Comment**

I am wondering whether the authors can provide further explanation about why they included only female caregivers. The authors stated, “Older people in the study setting are cared for predominantly by female family members, especially their daughters, in their private homes”. Does it have something to do with culture or religion? Or something else? Also, it would be good to justify the selection of the study setting.

**Response**

Older people in the study setting are cared for predominantly by female family members, especially their daughters, in their private homes. This role is influenced by cultural values putting expectation to women within the family to look after their parents.

**Comment**

Do the hospital or healthcare facilities in the study setting have specific services for older people during the COVID-19 pandemic?

**Response**

All the hospitals in study setting did not have specific services for older people during COVID-19 pandemic.

**Comment**
It would be helpful for the readers if the authors can provide some examples of the main research questions used to explore the topic.

**Response**

The interviews were guided by several predetermined main questions and probing questions were developed during the interview. Some examples of the main questions are “What actions did you take to protect older people or parents during COVID-19 pandemic? What challenges have you experienced when protecting older people during COVID-19 pandemic? What is your experience about older people’s adherence to the actions taken to protect them from COVID-19? The decision about the questions was made through the process of formulation, discussion, and revision.

**Comment**
What are the implications of your findings for the health department or the government in the study setting? The authors mentioned intervention programs to support both female caregivers and older people. Can you propose any appropriate intervention programs that may have been implemented in other settings? Food support, nutritional supplement support, etc….

**Response**

The results of the study can contribute to developing intervention programs such as providing food support and nutritional supplements for female family caregivers and older people living at home in poor or limited resource settings.
